# Extrafloral-nectar-based partner manipulation in plant–ant relationships

**DOI:** 10.1093/aobpla/plv002

**Published:** 2015-01-14

**Authors:** D. A. Grasso, C. Pandolfi, N. Bazihizina, D. Nocentini, M. Nepi, S. Mancuso

**Affiliations:** 1 Department of Life Sciences, University of Parma, Viale delle Scienze 11/a, 43124 Parma, Italy; 2 LINV - Department of Agrifood Production and Environmental Sciences, University of Florence, Viale delle Idee 30, 50019 Sesto F.no, Florence, Italy; 3 Department of Life Science, University of Siena, Via P.A. Mattioli 4, 53100 Siena, Italy

**Keywords:** Ant–plant interactions, extrafloral nectar, manipulation, mutualism, myrmecophytes, secondary compounds

## Abstract

Plant–ant interactions are generally considered as mutualisms, with both parties gaining benefits from the association. It has recently emerged that some of these mutualistic associations have, however, evolved towards other forms of relationships and, in particular, that plants may manipulate their partner ants to make reciprocation more beneficial, thereby stabilizing the mutualism. Focusing on plants bearing extrafloral nectaries, we review recent studies and address three key questions: (i) how can plants attract potential partners and maintain their services; (ii) are there compounds in extrafloral nectar that could mediate partner manipulation; and (iii) are ants susceptible to such compounds? After reviewing the current knowledge on plant–ant associations, we propose a possible scenario where plant-derived chemicals, such as secondary metabolites, known to have an impact on animal brain, could have evolved in plants to attract and manipulate ant behaviour. This new viewpoint would place plant–animal interaction in a different ecological context, opening new ecological and neurobiological perspectives of drug seeking and use.

## Abstract (precis)

Many plant-derived chemicals may have an impact on the functioning of the animal brain. The mechanisms by which the psychoactive components of these various products have their effects have been widely described, but the question of why they have these effects has been almost totally ignored. Recent evidence suggests that plants may produce chemicals to manipulate their partner ants and to make reciprocation more beneficial. In the present review we propose that these plant-derived chemicals could have evolved in plants to attract and manipulate ant behaviour; this would place the plant–animal interaction in a different ecological context and open new ecological and neurobiological perspectives for drug seeking and use.

## Introduction

Interactions among organisms are receiving increasing attention both for their ecological implications and as an important interpretative tool in evolutionary biology, genetics, immunology, development and physiology (Stadler and Dixon 2008; Douglas 2010; Gilbert *et al*. 2012). In particular, plant–ant relationships offer an outstanding array of interactions, being both among the most diverse and dominant multicellular organisms on Earth that coevolved for over 100 million years (Mayer *et al*. 2014). This long common story, often characterized by coevolutionary pathways, has led to the development of many adaptations whose knowledge could be extremely important in many fields of basic and applicative biology (Schoonhoven *et al*. 2005; Thompson 2005, 2013). In particular, interactions between ants and plants provide numerous examples of mutualism (Janzen 1966; Rosumek *et al*. 2009) dating back to the mid-Cretaceous period (100 million years ago) when both parties had a dramatic diversification and radiation, often reciprocally influencing their evolutionary pathways (Wilson and Hölldobler 2005; Weber and Agrawal 2014).

In mutualistic plant–ant interactions, both parties gain benefits from the association (Davidson and McKey 1993; Byk and Del-Claro 2011). However, it has recently emerged that some of these associations have evolved to maximize plant-derived rewards through plant-driven ant manipulation (e.g. Heil *et al*. 2014). In the present review, we discuss the importance of the control exerted by plants on ant behaviour in their multifaceted interactions focusing on the extrafloral nectar (EFN), as EFN seems to be specially designed to influence and reward ants for their protective services. Starting from recent advances in the field, we bring forward several hypotheses on the putative role of secondary metabolites in EFNs in plant–ant relationships and identify key outstanding issues that need to be addressed to fully understand these fascinating associations.

## Plant–Ant Mutualistic Interactions

Ants (order: Hymenoptera; family: Formicidae) are dominant organisms in most terrestrial habitats having reached, among social insects, the most impressive adaptive radiation. The key to their success is their colonial life; the complexity of their social life allows ants to control their physical environment, exploit resources in an efficient way and overcome competitors (Hölldobler and Wilson 2009). Ants can establish a complex network of interactions with virtually every component of their ecosystems, ranging from microorganisms to fungi, and from other animals to plants (Ness *et al*. 2010). Interactions involving ants and plants constitute textbook examples, from antagonism and opportunism to occasional mutualism and obligate symbiosis. In addition, these interactions are geographically widespread and have been shown to be critical in shaping many ecosystems (for comprehensive reviews, see Buckley 1982; Beattie 1985; Jolivet 1986; Hölldobler and Wilson 1990, 2009; Huxley and Cutler 1991; Davidson and McKey 1993; Blüthgen *et al*. 2000; Heil and McKey 2003; Beattie and Hughes 2006; Blüthgen and Stork 2007; Rico-Grey and Oliveira 2007; Lange and Del-Claro 2014).

Plant–ant association evolved quite early, as already several ferns have various adaptations to ants, including rhizomes riddled with tunnels used as nesting site or nectariferous structures used as food source (Beattie 1985). However, it is in angiosperms where plant–ant relationships reached high levels of complexity and coadaptation. A classical example is that of myrmecophyte species that provide ants with: shelters in specialized organs (stems, leaves and spines) evolved as adaptation to facilitate ant nesting (domatia); different sorts of food bodies containing nutrients according to the species (proteins, lipids, glycogen) and extrafloral nectaries producing sugar-rich secretions and other compounds (Hölldobler and Wilson 1990; Rico-Grey and Oliveira 2007; Ness *et al*. 2010).

Mutualistic ants are generally considered as an efficient tool for plants' indirect defence against herbivores (e.g. Agrawal and Rutter 1998; Bronstein 1998; Heil and McKey 2003; Heil 2008). Several features of feeding ecology and social behaviour of ants make them ideal partner for defence purposes: they build stable nest, may patrol wide areas night and day, defend territories and often adopt efficient recruitment strategies towards place where abundant food or potential threats are located (Hölldobler and Wilson 1990). Furthermore, several ant species are both sugar collectors and robust predators, exhibiting very aggressive reactions against other animals that may represent a potential threat for their food resource and nesting sites (Rico-Grey and Oliveira 2007; Stadler and Dixon 2008; Cerdá and Dejean 2011). Obviously, in order to exploit these services and improve the quality and stability of the association, plants need to attract the ants by providing them with shelters and nutritionally rich food sources. In addition, evidence also indicates that EFN can significantly increase ant colony survivorship, growth and reproduction (Byk and Del-Claro 2011).

## Floral and Extrafloral Nectar Composition, and their Role in Plant–Animal Interaction

Nectars can be defined as a plant secretion mediating mutualistic interactions with a large array of animals, which, from an ecological point of view, can be divided into two main groups: pollinators rewarded with floral nectar and anti-herbivory defenders rewarded with EFN (Nicolson and Thornburg 2007; González-Teuber and Heil 2009*a*). The two types of nectars share the basic chemical composition, with simple carbohydrates, mainly glucose, fructose and sucrose, being the most abundant solutes; as both nectars are easily digested and absorbed they both fulfil the high-energy demands required to sustain animal activities (Escalante-Pérez and Heil 2012; Nepi *et al*. 2012; Nepi 2014*a*). In general, nectar contains a combination of these three sugars, although specialization in carbohydrate composition exists both in floral and EF nectars (Baker and Baker 1983*a*, *b*; Heil *et al*. 2005). For instance, hummingbirds specialize on nectar feeding, and being floral nectar their only alimentary resource, they have a high invertase activity in their digestive tract, which allow them to consume preferentially sucrose-dominant nectars (Nicolson and Thornburg 2007). On the other side, passerine birds, being opportunistic nectar feeders, possess generally a low invertase activity and are therefore obligated to consume hexose-dominant floral nectar (Nicolson and Thornburg 2007).

After sugars, amino acids are the more abundant compounds in nectars. Despite being, in general, 100–1000 times less concentrated than sugars, nectars have a primary alimentary importance as nitrogen source and protein constituents. Thus, in the case of EFN, the unbalanced carbon-to-nitrogen (C/N) ratio of the reward may increase ants’ desire for N-rich protein and hence the likelihood that they will attack herbivorous insects on the host plant, potentiating their indirect defence (Ness *et al*. 2009). Variations in this ratio can be induced by herbivore activities, with both EFN sucrose (Ness 2003) or amino acid (Smith *et al*. 1990) contents increasing following herbivore attacks. Interestingly, mixtures of sugars and amino acids, which mimic EFN after herbivore attack, have been found to be particularly attracting to ants (Lanza *et al*. 1993). Amino acids confer specific tastes to floral and EF nectars, affecting both their attractiveness (Blüthgen and Fiedler 2004; Nicolson and Thornburg 2007; Nepi *et al*. 2012; Nocentini *et al*. 2013) and their alimentary importance, mostly in animals for which nectar is the only food resource. Accordingly, the concentration of amino acids is generally higher in floral nectar consumed by insects compared with that consumed by birds or bats that do not feed exclusively on nectar (González-Teuber and Heil 2009*a*; Escalante-Pérez and Heil 2012). Specific behavioural responses to different amino acids are known for floral and EF nectar consumers. For instance, proline, which is one of the amino acids preferred by bees (Bertazzini *et al*. 2010), is also one of the most abundant amino acids in the floral nectar of several melittophilous plants (Nocentini *et al*. 2012). Preferences for specific amino acids or for specific mixtures of amino acids are known also for ants, and can vary among ant species depending on their nutritive needs and their specialization (e.g. myrmecophyte *vs.* non-myrmecophytes species, Blüthgen and Fiedler 2004; González-Teuber and Heil 2009*b*). For example, behavioural assays with obligate *Acacia* inhabitants and non-symbiotic ants showed that symbiotic and non-symbiotic ants differ in their preferences for artificial amino acid solutions; in the non-symbiotic ants, just the presence of amino acids in the nectar was found to be important but not their detailed identity, while symbiotic ants were found to be much more selective (González-Teuber and Heil 2009*b*). Indeed, symbiotic ants were able to distinguish a specific solution containing four specific amino acids (leucine, phenylalanine, proline and valine), highly concentrated in the EFN of their host plant (*Acacia hindsii* Benth.), from solutions containing other amino acid mixtures; these results suggest that not only amino acid concentrations but also their number and detailed identity played a key role in preferences by symbiotic ants.

## Ants and Plants Bearing Extrafloral Nectaries: A Special Case

**Figure 1.**
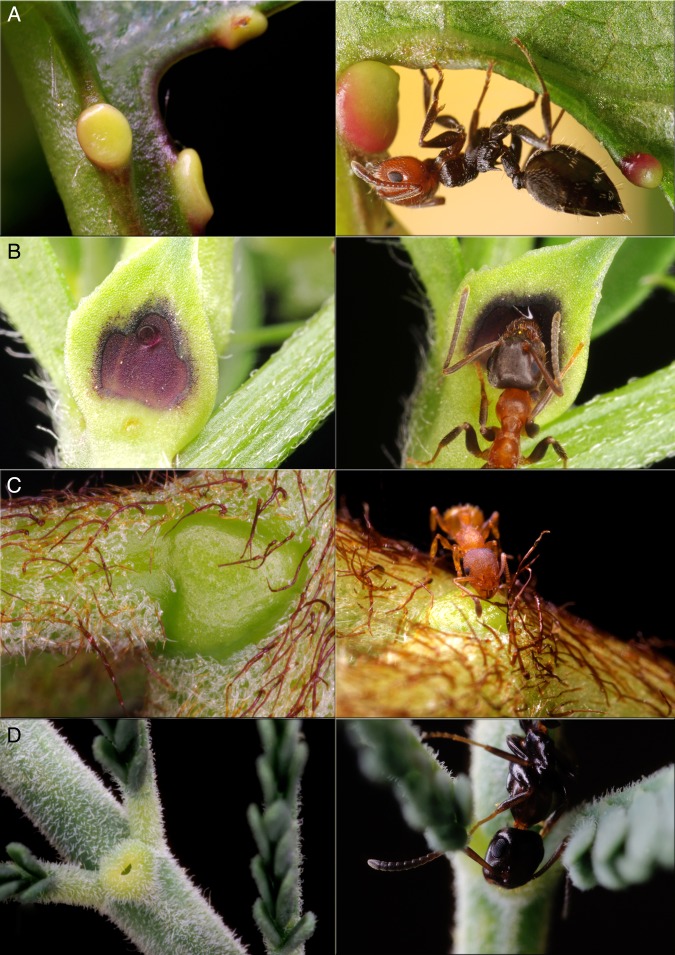
Extrafloral nectaries of different plants (left) and visiting ants (right). (A) *Prunus avium*, *Crematogaster scutellaris*; (B) *Vicia sativa*, *Lasius emarginatus*; (C) *Pteridium aquilinum*, *Temnothorax parvulus*; (D) *Acacia dealbata*, *Camponotus truncatus* (pictures by D. Giannetti and D.A. Grasso, Myrmecology Lab, University of Parma).

A large number of plants bear nectaries that are not associated with reproductive functions but are mainly devoted to attract ants and other arthropods (Marazzi *et al*. 2013; Fig. 1). Since the pioneering studies and vivid descriptions by (Delpino 1874, but see also Mancuso 2010), numerous studies on these structures and their possible biological roles have greatly extended our understanding of plant–ant interactions. Extrafloral nectaries are common and widespread in many vascular plants and generally considered as a tool used by plants to attract animals for defensive purposes. Extrafloral nectaries consist of glands producing nectary secretions associated with vegetative structures as leaves, stems and stipules, which are however, as above stated, not linked to pollination. Although EFN has been found to attract different species of insects and arthropods (e.g. parasitoids, predatory mites, spiders), in most cases it seems to be specially designed to attract ants, whose feeding ecology and behaviour fit very well the plant's defensive needs (Beattie 1985; Schoonhoven *et al*. 2005; Heil 2008).

Extrafloral nectaries are a highly diverse, evolutionary-labile and phylogenetically widespread plant traits. They have been reported in about 4000 plant species (see at http://biosci-labs.unl.edu/Emeriti/keeler/extrafloral/worldlistfamilies.htm and Table 1), but according to Weber and Keeler (2013) the estimated number of species having EF nectaries could be much higher, up to an estimated 8000 or more species. As in angiosperms there are different degrees of interactions with ants, it is plausible that the evolution of mutually beneficial traits, such as EFNs, drove the diversification of both angiosperms and ants during middle Cretaceous when both groups of organism were radiating (Rico-Grey and Oliveira 2007; Marazzi and Sanderson 2010). Since ants are rarely efficient pollinators, whilst more frequently they are nectar thieves (Ballantyne and Willmer 2012), it has been suggested that, in plants, EFN may have evolved as an attractive device to keep ants away from the floral nectar (Wagner and Key 2002). On the other hand, there are now many convincing studies that demonstrate the importance of EFN as a tool to attract ants as effective agents against herbivores or plant competitors (see Koptur 1992; Heil 2008). The defensive effect of ants is so conspicuous that there is a long history of using these animals as biocontrol agents (Heil 2008) and there are now numerous studies that report plant protection by ants in a wide range of habitats, from temperate to tropical environments (reviewed in Heil and McKey 2003; Rico-Grey and Oliveira 2007).

**Table 1.** Frequencies and functions of EFN in terrestrial plants. ^1^From http://biosci-labs.unl.edu/Emeriti/keeler/extrafloral/worldlistfamilies.htm. ^2^Gnetophyta produce nectar close to the reproductive structures and its function is ecologically more similar to floral nectar.

Plant group	Number of species with EFN/% on total number of species^1^	Functions	References
Bryophyta	0	–	
Pteridophyta	44–126/0.3–0.9 %	Anti-herbivory defence (not firmly confirmed)	See Koptur *et al*. (2013)
		Spore-dispersal	Koptur *et al*. (1982); Tryon (1985)
Spermatophyta			
Gymnosperms	Few Gnetophyta^2^	Reward for pollinating insects	Nepi *et al*. (2009)
Angiosperms	4212/1.7 %	Anti-herbivory defence	See Rico-Grey and Oliveira (2007)
	Particularly frequent in Fabaceae, Passifloraceae and Malvaceae	Luring ants from floral nectar	Becerra and Venable (1991)
		Distracting ants from tending hemipterans	Becerra and Venable (1989)

Ants can reduce the cost of herbivory by deterring or preying upon insects and vertebrate herbivores. In most cases, the mere ant presence during patrolling can dislodge or frighten away plant enemies, or harass them during feeding, egg laying, courtship or molting, with a strong beneficial impact on plant fitness (Beattie 1985). Recently, a remarkable case study also established a clear link between ant leaf patrolling activities and leaf protection against pathogens in the myrmecophyte *A. hindsii* (González-Teuber *et al*. 2014). In plants inhabited by ants, mutualistic ants substantially reduced pathogen-inflicted leaf damage and epiphytic bacterial abundance compared with parasitic ants; this beneficial effect of mutualistic ants was associated with the presence of specific bacterial community on the ant's legs, including representatives of the genera *Bacillus, Lactococcus*, *Pantoea* and *Burkholderia* (González-Teuber *et al*. 2014). On the other hand, it has also been shown that the consumption of carbohydrate-rich EFNs increases the incentives for omnivores (i.e. ants) to act as carnivores, thereby leading to an increased aggressiveness against potential prey (i.e. herbivores; Ness *et al*. 2009). Finally, in a study conducted in *Acacia drepanolobium*, it emerged that the presence of mutualistic species could provide a direct metabolic benefit to the plants that, in turn, enhanced the pool of photosynthates available for additional defence and/or for tolerance-related growth (King and Caylor 2010). Indeed, in the presence of the strong mutualistic ant species, *Crematogaster mimosae* and *C. nigriceps*, the net leaf photosynthetic rate of the trees increased by up to 30 % compared with plants with no patrolling ants; as the mutualistic ants eat part of the host trees' axillary and terminal shoots, this photosynthetic up-regulation was likely to be associated with the ant-induced tissue loss and damage (King and Caylor 2010).

Mutualisms are prone to exploitation by low-quality symbionts that do not provide an adequate service to their host (e.g. King and Caylor 2010; Heil 2013), and this raises the question of whether plant can actively sense the presence of ants and monitor their activity and their identity (e.g. parasitic and mutualistic ants, cf*.* Heil 2009, 2013). Indeed, although partner choice (that entails the host identification of the future partner) and host sanctions (that requires host monitoring of the quality of the service provided by the partner) have been considered as effective mechanisms to stabilize mutualisms (Bull and Rice 1991; West *et al.* 2002), both strategies require for the hosts to be able to directly judge the identity or the actions of their partners (Heil 2013). In the simplest model, plants could assure the preferred association with mutualistic ants simply through a ‘competition-based screening’ that can occur both before and after the initial colonization process (Heil 2013). As mutualistic ants are more adapted than the parasitic ones to make use of the plant-derived food sources, and increased EFN secretion rates increase ant activity and aggressiveness, then higher EFN production would favour mutualistic rather than parasitic ants in a closed loop of positive feedback mechanisms between ant activity and EFN production (Bixenmann *et al*. 2011; Heil 2013). Hence, in this context, plants could monitor the identity and activity of ants without the need for active sensing and monitoring (Heil 2013). Nevertheless, this hypothesis does not fully answer the question of whether plants can actively assess ants' identity and activity, and further investigations are required to bring forward alternative hypotheses regarding specific mechanisms (e.g. chemical, mechanical or electrical cues) by which this could be achieved.

## EFN-Bearing Plants Interactions with Ants: How to Attract a Partner (and Maintain its Services)?

**Figure 2.**
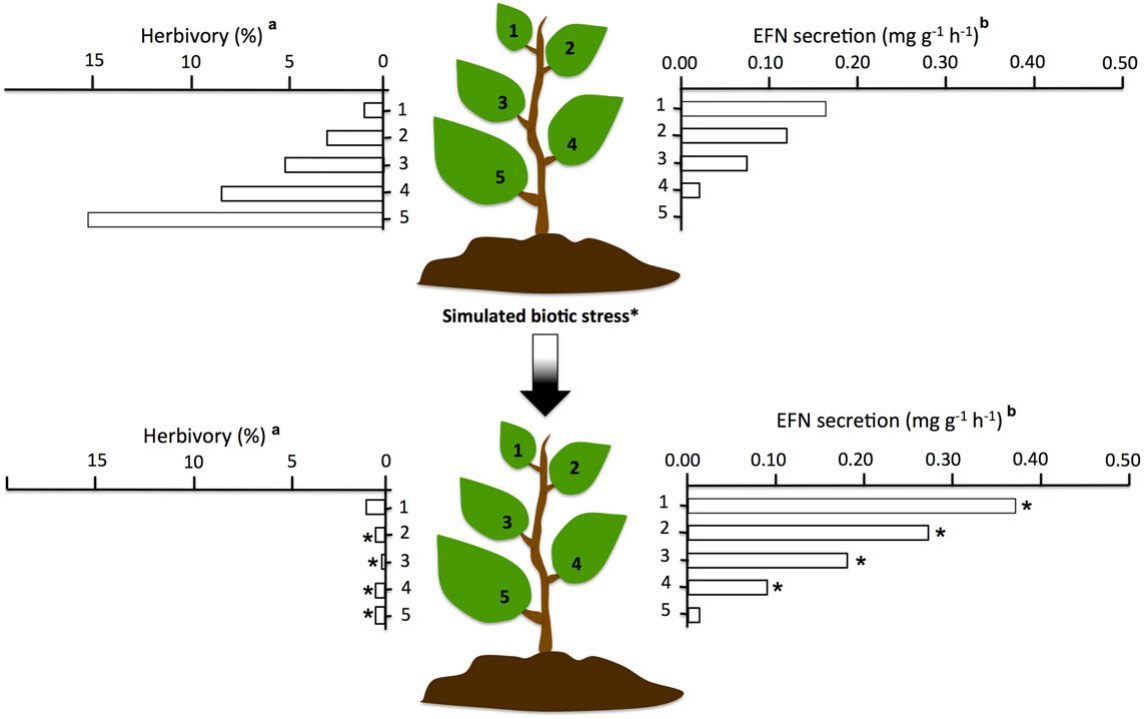
Herbivory and EFN secretion rates in differently-aged leaves before and after treatment with jasmonic acid, a hormone that can be used to simulate a herbivore attack on plants. ^a^Data modified from Heil *et al*. (2001). ^b^Data modified from Millán-Cañongo *et al*. (2014).

**Figure 3.**
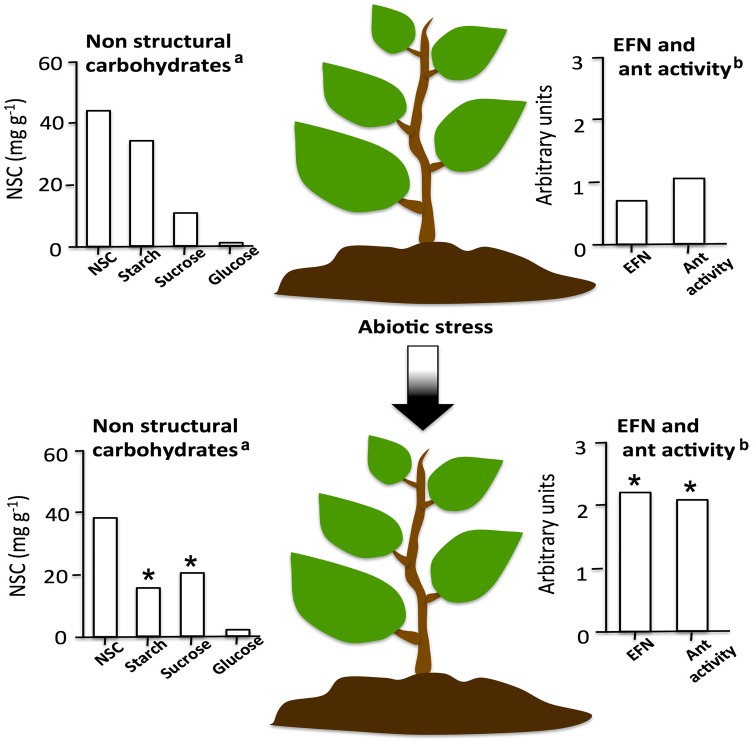
Extrafloral nectar secretion rates, ant activity and non-structural carbohydrate (NSC) composition of EFN under control conditions or following a drought stress. ^a^Data modified from Pringle *et al*. (2013). ^b^Data modified from González-Teuber *et al*. (2012).

The activity of EF nectaries can be constitutive, i.e. nectar is permanently secreted, or can be induced. Induction has been widely documented in temperate plants but has not been reported in tropical plants, where higher and more constant herbivore pressure would favour constitutive defences rather than induced defence (Bixenmann *et al*. 2011). When inducible, nectar secretion can be modulated by the presence of herbivores and/or of ants and by abiotic stresses (Heil *et al*. 2001; Heil 2004; Wooley *et al*. 2007; Bixenmann *et al*. 2011; Millán-Cañongo *et al*. 2014; Figs 2 and 3). Evidence suggest that higher host's investment in EFN can be associated with better protection against herbivores as higher EFN production have been found to favour mutualistic ants relatively more than parasitic ones that, as above stated, despite using of the plant-derived rewards, do not give back an adequate service (Baker-Meio and Marquis 2012; González-Teuber *et al*. 2012; Heil 2013). Recent comparative studies of Mesoamerican *Acacia* myrmecophytes, characterized by different levels of host reward, provided further evidence supporting this hypothesis (e.g. Heil *et al*. 2009; Heil 2013). Indeed, more than 90 % of the high-reward plants were found to be occupied by mutualistic ant species, whereas only 50 % of the low-reward hosts were inhabited by parasitic, i.e. non-defending, ant species (Heil *et al*. 2009). Subsequently, upon monitoring, for 7 months, one high and low-reward species, it was found that EFN secretion levels was a strong predictor of occupancy by mutualistic or parasitic ants, with greater EFN levels associated with higher occupancy by mutualistic ant species (Heil 2013). Interestingly, plant protection is improved not only by a higher ant visitation rate but also by an increase in the active search for protein sources (e.g. herbivorous insects, i.e. aggression) resulting from dietary imbalances imposed by the sugar-rich EFN (Ness *et al*. 2009).

A key point in all forms of indirect defence is how to attract the attention of potential partners in order to get their services. In the context of the partnership between ants and plants bearing EF nectaries, secretions produced by nectaries function themselves as attractants, as carbohydrates and amino acids are a fundamental component of their diet (Blüthgen and Fiedler 2004; Detrain and Prieur 2014). These two primary classes of nectar solutes (sugars and amino acids), as well as their relative proportions, determine insect's feeding behaviour, as they influence taste and respond to specific alimentary requirements. Thus, changes in the chemical profile of these two major classes of substances may induce differences in the attractiveness of nectar. However, in the perspective that nectar mediates interactions far more complex than simple alimentary relations, it is also important to pay attention to less-abundant compounds, not necessarily linked with the alimentary needs of insects. Indeed, although nectar is composed primarily of sugars, it has now been recognized that minor nectar constituents, such as secondary metabolites, can be crucial in mediating plant interactions with other species (Adler and Irwin 2012). Although there are major knowledge gaps regarding the presence of secondary metabolites in EFN, and much more is known for floral nectar, it is clear that secondary compounds have a crucial role in regulating nectar-based interactions (Nepi 2014*a*); it is therefore plausible that, in addition to carbohydrates and amino acids, they may play a significant role as a tool to influence EFN-mediated ants' behaviour. In the context of plant–insect relationships, secondary compounds may have different effects from attraction to deterrence. For instance, in nectars, secondary metabolites such as caffeine have been found to elicit a range of behavioural and physiological responses, from attraction to avoidance and positive to negative response, depending on the secondary metabolite identity and/or concentration (Manson *et al*. 2013).

## Secondary Compounds: Nectar-Mediated Manipulation?

Despite very little is known about their ecological roles in nectars, it was recently proved that secondary compounds could affect the behaviour of nectar feeders' pollinators (Singaravelan *et al*. 2005; Kessler and Baldwin 2007; Raguso 2009; Wright *et al*. 2013; Nepi 2014*a*). Several secondary metabolites have been found in the nectar of species from at least 21 families (reviewed in Adler 2000) and, although their concentrations vary widely among plant parts, in nectars their concentration is generally lower compared with other plant tissue (Manson *et al*. 2012; Cook *et al*. 2013). Although many secondary compounds may affect animal behaviour, currently the main compounds thought to influence animal behaviour are non-protein amino acids (NPAAs) and alkaloids.

Non-protein amino acids, whose ecological significance is currently unknown, are a class of secondary compounds that has long been known to occur in nectar (Baker *et al*. 1978). Approximately 250 NPAAs have been found in plants, but only few of them in nectars, with the most common being GABA (γ-amino butyric acid), followed by β-alanine and then taurine (Nepi 2014*b*). Although most of the determinations referred to floral nectar, NPAAs have been reported to be more frequent in EFN rather than in floral nectar (Baker *et al*. 1978). Non-protein amino acids may have important roles in modulating the behaviour of nectar feeders, mainly in three different ways (Nepi 2014*b*).

1. They can directly influence the activity of the insect nervous system. Indeed, GABA, taurine and β-alanine are abundant in the nervous systems of animals, where they regulate neuronal excitability and thus behaviour. γ-Amino butyric acid is the principal inhibitory neurotransmitter in both vertebrates and invertebrates (Breer and Heilgenberg 1985). γ-Amino butyric acid receptors in invertebrates are located peripherally in muscle tissue and neuromuscular junctions, where they are bathed in haemolymph (Bown *et al*. 2006) and are thus more sensitive to changes in GABA eventually caused by GABA-rich nectar feeding.
2. γ-Amino butyric acid has a fagostimulant activity since it can stimulate the taste chemoreceptors of insects sensitive to sugars, thus increasing the feeding rate (Schoonhoven *et al*. 2005).
3. Non-protein amino acids may promote muscle performance of insects. β-Alanine, taurine and GABA are used by athletes to increase their performance and reduce fatigue (Nepi 2014*b* and references therein). Unfortunately, no study has yet investigated the effect of these compounds on the muscle activity of insects.

Alkaloids present in the nectars are thought to affect plant–animal interactions. Although, in some cases, relatively high concentrations have been found in nectars with toxic effects on insect foragers (González-Teuber and Heil 2009*a*, and references therein), these compounds often occur in nectar at low concentration (generally below those found in other plant parts, Manson *et al*. 2012; Cook *et al*. 2013). Remarkably, depending on their identity and/or concentrations, they can interact with animal brain functions, thereby modulating insect behaviour (Manson *et al*. 2013). For instance, it was discovered that the presence of nicotine, a typical insect-repelling alkaloid, is necessary to optimize the time spent on a flower by a forager, i.e. the number of flower visitors per aliquot volume of nectar produced; this enables plants to minimize nectar volumes while maximizing the transfer of pollen and seed production (Kessler and Baldwin 2007). In another example, low concentrations of nicotine and caffeine (both typical insect-repelling compounds) elicited a significant feeding preference in free-flying honeybees (Singaravelan *et al*. 2005). It was subsequently proposed that the presence of psychoactive alkaloids in nectar may lead to a dependence or addiction in pollinators, as well as improving the short-term and early, long-term memory of honeybees (Singaravelan 2010). Confirming this hypothesis, it was recently demonstrated that honeybees rewarded with solutions containing nectar levels of caffeine were able to remember a learned floral scent better than honeybees rewarded with sucrose alone (Wright *et al*. 2013). Caffeine's influence on cognition in bees is mediated by its action on the Kenyon cells in the mushroom bodies, and exhibit potentiation in associative learning (Heisenberg 2003), similarly to what happens in the hippocampal neurons of mammals. Furthermore, caffeine concentrations in nectar did not exceed the bees’ bitter taste threshold, implying that pollinators impose selection for nectar that is pharmacologically active but not repellent. Therefore, by using a drug to enhance memories of reward, plants can secure pollinator fidelity and improve reproductive success (Wright *et al*. 2013). Interestingly, it was demonstrated that leaf herbivory may increase the levels of alkaloids in nectar and thus interfere with foraging activity of the feeders (Adler *et al*. 2006). Unfortunately, this was demonstrated only for floral nectar and no information is available for EFN, although it is known that herbivory induces a systemic increase of deterrent molecules in all the plant body (Heil 2008). There is a general lack of studies concerning secondary compounds in EFN but recently Cardoso-Gustavson *et al*. (2013) reported trace amounts of alkaloids in the EFN of *Passiflora*. According to the authors, the amounts of alkaloids were not sufficient to cause deleterious effect to insect metabolism and growth and thus may have other functions than deterrence.

## Plant–Ant Interaction: Is there Room for Partner Manipulation?

The reciprocal exchange of benefits is the key feature of mutualistic interactions but benefits are often costly to provide, which then leads conflict among partners. These conflicts can be managed by a single controlling organism that may selectively reward cooperative partners and sanction to non-cooperative ones, control partner behaviour and eventually employ recognition mechanisms that discriminate between beneficial and potentially harmful or ineffective partners (Douglas 2010). All these mechanisms have been proposed to explain how ant–plant partnership may be stable under the danger of cheaters (Heil and McKey 2003). In this context, ant–plant interactions represent useful and promising study models for interdisciplinary investigations (involving ethology, behavioural ecology, neurophysiology, plant biology and physiology, evolutionary biology).

## Are Ants Vulnerable to Manipulation?

In spite of their ecological dominance and superorganismic efficient organization, ants are vulnerable to manipulation by other organisms. A plethora of parasites, exploiters and cheaters have been found to affect ant anatomy, neurophysiology and behaviour (Hölldobler and Wilson 1990; Hughes 2012). For instance, nematode infections have been found to alter the anatomy and behaviour of the parasitized ants so that it resembles a ripe fruit to be dispersed by birds (Hughes *et al*. 2008). Another amazing example of a parasite-extended phenotype is that of the death grip in ants, observed when *Ophiocordyceps* fungi infected them to facilitate spore dispersal (de Bekker *et al*. 2014). Ants also suffer social parasitism by several insects and other ants (Hölldobler and Wilson 1990), and in this case, the host behaviour may be strongly manipulated by the parasite. For example, raiders of slave-making ants have been found to use chemicals to cause panic inside attacked colonies, and queens discharge appeasement substances to lower the level of aggression in resident workers during host colony usurpation (Mori *et al*. 2001).

Interaction between ants and plants bearing EF nectars may result in aggressive behaviour upon encounters with intruders. Indeed, most of the ant species common on EF nectaries are rather aggressive showing strong ownership behaviour and fierce attack responses against intruders (Bentley 1977). More specifically, as reported by Koptur (1992), ants' discovery of EFN resources induces the following behavioural repertoire: feeding, collecting, recruitment, territoriality and aggression. Aggressive behaviour is particularly evident in myrmecophyte–ant associations. For example in *Tetraponera–Barteria* association, ants are extremely aggressive and prone to respond to fine vibration perceived on the plant; this results in ants attacking insects and even large mammalian herbivores (even elephants and antelopes) approaching the plant, and in extreme cases ants can drop down the tree to attack and sting painfully the intruders. Finally, aggressive ants can also emit a strong-smelling secretion that may serve as a warning signal to approaching animals (Janzen 1972; Dejean *et al*. 2008). Ants are also very active against encroaching vegetation. This aggression against competing vegetation is noteworthy since ants normally attack other invertebrates (enemies or prey) or vertebrates perceived as threat for the colony. In this case, it is likely that ants are particularly sensible to some chemical or mechanical stimuli from the plants, which then elicits their ‘allelopathic’ aggression. These behaviours probably derive, as specialized forms, from predatory or nest cleaning behavioural patterns as that showed by some *Formica* and *Pogonomyrmex* spp. (Beattie 1985).

However, not all ants behave in the same way and some species are better than others in protecting their plant partners (Koptur 1992). Therefore, a strong selection is expected on plants to get the best they can among the potential partners and avoid any form of exploitation of their mutualistic habit (Orona-Tamayo and Heil 2013). One of the best-studied example is that of *Acacia* and their obligate *Pseudomyrmex* partners, which feed only on the sucrose-free nectar produced by their host that is not attractive for generalist exploiters (Heil *et al*. 2014). This ‘specialization’, however, hides an amazing case of partner manipulation by the host plant. In fact, invertase (sucrose hydrolytic) activity is not constitutionally absent in the ant midgut but is inhibited by chitinase, a dominant EFN protein. Once enclosed, young workers ingest EFN as the first diet available and their invertase becomes inhibited. In this way, they are forced to continue feeding on host-derived EFN being unable to digest any other food. In this ant–acacia mutualism, the plant manipulates the digestive capacities of the symbiotic ants to enhance their dependence on the host-derived food rewards, thus stabilizing in this way the partnership and avoiding possible interference by exploiters. To our knowledge, this is the first clear example of partner manipulation in a plant–ant mutualism based on EFN secretion, as it represents a dramatic change which appears disadvantageous for the ant, at least when considering its possibilities to return to a free-living life style.

It could be expected that the above-mentioned example is not the only case of partner manipulation in a plant–ant mutualism as EFNs may have important manipulative and direct effects on other aspects of ant biology and behaviour. Under the manipulative hypothesis, it is possible that some of the secondary nectar components (already known or still to identify, see previous sections) have significant effects on ant physiological and behavioural traits resulting in a more effective service for the plant. In this context, it is worth noting that EFN secretion, or amino acid concentrations in EFN, may increase in response to herbivory, and so nectar composition could be tuned with the actual defensive needs of the plant to acquire better services by the ants (Heil and McKey 2003; Heil 2008; González-Teuber and Heil 2009*b*; Shenoy *et al*. 2012; Fig. 2). The core of indirect defence by mutualism with ants is defence against enemies. Hence, among the most obvious aspects of ant behaviour potentially affected by plant manipulation, there is aggression. Indeed, as stated above, ants associated with plants (especially myrmecophytes) are generally extremely aggressive and reactive to intruders and even alien not living objects. Thus, it is plausible that EFN-mediated manipulation can affect aggressiveness.

Extrafloral nectar could affect several other aspects of ant biology, not necessarily linked with increasing aggression that could promote ant defensive or protective effects. Most ant–plant mutualisms are facultative or, in some cases, occasional associations, and involve groups of species that may vary in time, space and impact on plant fitness (Bronstein *et al*. 2006). In facultative ant–plant mutualism, the mere presence of ants has been found to exert significant effects on plant performance due to non-consumptive effects that deter significantly plant predators or dramatically affect their behaviour with a beneficial cascade effect on plant fitness. For example, in *Gossypium thurberi*, the associated ants (*Forelius pruinosus*) have a strong disturbing effect on the folivore caterpillars that alter their behaviour, thus reducing plant damage (Rudgers *et al*. 2003). Finally, there are experimental evidences showing that the nutritional composition of EFN can alter foraging preferences of ants, enabling plants to manipulate the prey preferences of their mutualistic partners; in this way, plants could ultimately bias prey selection of the ants towards herbivores, competitors or predators that pose the greatest risk to the plant (Wilder and Eubanks 2010).

Inconspicuous actions may also be expression of other important defensive services that ant partner may offer, such as cleaning and protection against pathogens and fungi (Beattie 1985; Rico-Grey and Oliveira 2007; Heil 2008). Interestingly, even inconspicuous actions by non-aggressive species may have crucial protective effects on plants, as in the case of *Pheidole bicornis*, a small and sluggish ant associated to *Piper* spp.; workers of these species clean the surface of leaves from eggs and early instar larvae of herbivore insects, thereby significantly reducing their negative impact on plants (Letourneau 1983). Hence, in order to record significant effects on the plant fitness, it is not necessary to imagine dramatic and substantial changes in ant behaviour due to eventual plant manipulation strategies. In accordance with the manipulative hypothesis, it has recently been shown that four alkaloids such as caffeine, theophylline, cocaine and atropine investigation can have significant effects on many aspects of ant physiology and behaviour (Cammaerts *et al*. 2014). In particular, feeding on the alkaloids altered locomotion, memory, olfactory perception and reactions to stimuli in the model ants (*Myrmica sabuleti*); in the case of cocaine, dependence was also recorded (Cammaerts *et al*. 2014). Interestingly, in other investigation, morphine addiction in ants was reported, as well as behavioural effects on memory and learning. Morphine administration activates the dopamine reward pathways and affects serotonin expression (Entler *et al*. 2012).

The recent recognition that minor nectar constituents (e.g. secondary metabolites) are crucial in plant interactions with other species highlights the utmost importance of clarifying the neurophysiological and behavioural effects of possible neuro-active compounds present in EFNs as a manipulator system. Increasing attention has being devoted on these aspects (ant brain anatomy and neurophysiological aspects of ant behaviour) thanks also to the improved analytical techniques (see for example Gronenberg 2008; Penick *et al*. 2014; Pfeiffer and Homberg 2014). Further investigation in the context of ant–plant interactions will surely add more insight into this interesting topic with possible extensions to more complex neurophysiological systems and eventual applicative outputs on behavioural and physiological manipulation of animals by plants.

## Conclusions

**Figure 4.**
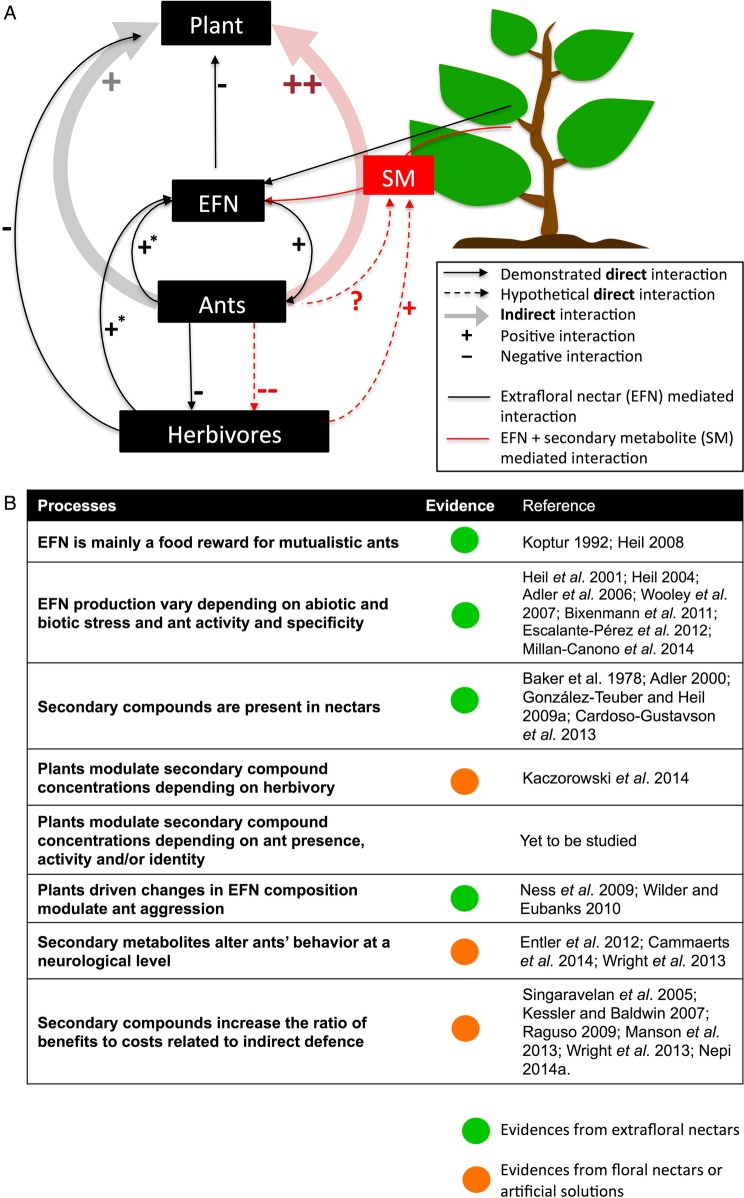
Representations of demonstrated and hypothetical plant–ant interactions. (A) Conceptual diagram representing ecological processes in plant–ant interactions. For clarity, the diagram is limited on the interactions that could be affected by secondary metabolites, and many other interactions (e.g. interactions mediated by volatile compounds) have been omitted. The negative interaction between EFN production and plant refers to the fact that, despite being considered a cost-efficient defence strategy in the presence of herbivores, EFN production is costly for plants (Bixenmann *et al*. 2011). (B) Critical processes involved in plant-driven ant manipulation and key knowledge gaps that require investigation in plants bearing extrafloral nectaries. SM, secondary metabolites; EFN, extrafloral nectar; * for temperate plants where the activity of EF nectaries is inducible; ?, due to the lack of studies it is not possible to bring forward an hypothesis on whether the interaction is positive or negative.

The indirect herbivore defence service that ants provide for some plants is a fascinating relationship. Indeed a key point in the definition of mutualism is that both involved parties may gain benefits from the association. Mutualism, however, typically imply costs for one or both partners and in a dynamic (co)evolutionary scenario, it is expected that each partner maximizes the benefits and minimize their costs. In this context, a relevant role could be played by contingencies and environmental constraints, and the different outcomes may vary in time and space (Menzel *et al*. 2014). This is particularly likely in diffuse interactions, where multiple species can associate with each other, as is the case of facultative associations between plants bearing EF nectaries and ants. In the present review, we bring forward the hypothesis that plants could maximize ant-derived rewards (i.e. defence) through secondary metabolite-mediated ant manipulation (see Fig. 4). In this case, we propose that ant manipulation through secondary metabolites could be a possible mechanism to stabilize mutualism by controlling/manipulating the partner (i.e. the ant). Indeed current theory on mutualism predicts that cooperation between organisms is evolutionarily unstable in the absence of mechanisms that counteract the temptation to cheat (Bronstein 2001; Frederickson 2013). From the perspective of each partner, a successful mutualism will maximize the ratio of benefits to costs and be minimally susceptible to cheating; thus, as already hypothesized for carbohydrate-rich EFN (Ness *et al*. 2009), from a plant perspective, EFN rewards with specific secondary metabolites may fulfil these requirements as it could reduce or maintained EFN costs but yield the most effective reward. Clearly detailed studies of the costs (from both the ant and plant perspective) associated with the proposed plant manipulation strategies via secondary metabolites are of outmost importance to fill all the outstanding issues.

To conclude, given that some plant-derived chemicals (i.e. secondary metabolites) have an impact on the functioning of the animal brain, we propose that many neuro-active compounds produced by plants evolved, not as a mere deterrent for animals, but also as a tool to attract and manipulate animal behaviour. However, despite there is evidence that secondary metabolites can influence ant behaviour, specifically preference and memory, there is still quite large knowledge gaps that need to be filled in order to fully understand the nature of plant–ant mutualistic associations (see Fig. 4). This new viewpoint would place plant–animal interaction in a different ecological context and open many new ecological and neurobiological perspectives of drug seeking and use.

## Sources of Funding

This work was supported by the following funds: grant to S.M. from the Italian MIUR PRIN Project PRO-ROOT; grant to C.P. from the FP7-PEOPLE-2012-IEF - n° 326202.

## Contributions by the Authors

S.M. and D.A.G. conceived the idea behind the review; D.A.G., M.N., C.P., N.B. and D.N. drafted the article and all authors commented on the draft.

## Conflicts of Interest Statement

None declared.
